# Neurotrauma pediatric scales

**Published:** 2008-11-15

**Authors:** Vlad Ciurea Alexandru, Mihaela Sandu Aurelia, Popescu Mihai, Mircea Iencean Stefan, Davidescu Bogdan

**Affiliations:** * Emergency Clinical Hospital Bagdasar-Arseni, 1st Department of General Surgery, UMF “Carol Davila”, Bucharest; ** Emergency Clinical Hospital Bagdasar-Arseni, 1st Department of Neurosurgery, Bucharest; *** District Hospital Pitesti, Department of Neurosurgery, Pitesti; **** Emergency Clinical Hospital Prof. Dr. N. Oblu, Iasi; ***** Universitary Hospital Constanta, UMF “Ovidius”, Constanta

**Keywords:** TBI, children, infants, toddler, neurotrauma pediatric scales, Pediatric Coma Scale, Children’s Coma Score, Trauma Infant Neurological Score, Glasgow Coma Scale, Liege Scale, neurotrauma outcome scales, Glasgow Outcome Scale, Glasgow Outcome Scale, KOSCHI score, Barthel Index, Head Injury Severity Scale

## Abstract

Cranial traumas have different particularities in infants, toddlers, preschool child, school child and teenagers. The assessment of these cases must be individualized according to age. It is completely different in children that in adults. Trauma scales, very useful in grading the severity and predicting outcome in traumatic brain injury, used in adults must be adapted in children. Children have age-related specificity and anatomic particularities, for each of this period of development. Neurotrauma scales, specific for infants and children, such as Pediatric Coma Scale, Children’s Coma Score, Trauma Infant Neurological Score, Glasgow Coma Scale, Liege Scale are reviewed, as well as neurotrauma outcome scales, like Glasgow Outcome Scale, modified Rankin score, KOSCHI score and Barthel Index. The authors present these scales in an exhaustive manner for thoroughgoing pediatric neurotrauma standards.

## Introduction

Head injury is the leading cause of death and disability in children. Statistical analyses shows that almost half of patients with a head injury (HI) each year in the United Kingdom are children under 16 years, and approximately one third of the patients with cranial trauma per year in the United States are children aged between 0 and 14 years old.[**1**;**2**]

Most common causes of head injury are: falls, child abuse, sport accidents, assaults and motor vehicle accidents. According to age distribution of head injury, two risk groups are identified: 0-4 years old and 15-19 years old. Boys are affected by head traumas twice the rate of girls.[**1**]

Cranial traumas in children are represented by scalp, skull and/or brain injuries, resulting from a traumatic etiology (external physical force, rapid acceleration or deceleration of the head).

## Particularities of traumatic brain injury in children

Growing and development process from infant-toddler to teenager implies important, general and specific, anatomical and functional particularities of central nervous system (CNS). In each phase of development, there is a distinctive response to external damaging factors. Because of these age-related particularities, there is an immediate posttraumatic response completely different in children than in adults. 

Although children tolerate larger space-occupying, traumatic or nontraumatic, lesions than adults, consequences are similar.

Physical exam of a child with traumatic brain injury (TBI) must be quick and safe, and consists of several steps:

• Assessment of airway, breathing and circulation (ABC exam)

• Checking for signs of cervical spine cord injury (rare in young children), and cervical spinal immobilization 

• Evaluation of level of consciousness, pupils’ size, and their reaction to light

• Assessment of local traumatic injuries

• Neurological exam, calculating trauma scores, according to trauma scales used into respective neurosurgical department 

• General physical exam, and in politraumas calculating MISS (*Modified Injury Severity Scale*)

• Establishment of useful paraclinical tests

Neurologic exam of the child must be individualized according to the age and level of consciousness.

## Neurotrauma pediatric scales

In children we must use pediatric scales, according to age. The most important neurotrauma pediatric scales are: Pediatric Coma Scale (PCS), Children’s Coma Score (CCS), Trauma Infant Neurological Score (TINS), Glasgow Coma Scale (GCS) and Liege Scale.

***1. Pediatric Coma Scale/Children Coma Scale (PCS)(Simpson & Reilly, 1982)[**3**]***

This scale evaluates eyes opening response, verbal response and motor response to stimuli. 

**Table:** 

Eyes opening	Score
Spontaneously	4
To speech	3
To pain	2
None	1
Verbal response	Score
Orientated	5
Words	4
Vocal sounds	3
Cries	2
None	1
Motor response	Score
Obeys command	5
Localizes pain	4
Flexion to pain	3
Extension to pain	2
None	1

**Normal scores** differ according to age.

**Table:** 

0-6 months	9
6-12 months	11
1-2 years	12
2-5 years	13
> 5 years	14

PCS is used in evaluation of brain injury severity in preverbal children. Scores must be adjusted according to child’s age: 

• During the first 6 months, best verbal response is crying, so normal verbal score expected is 2, and best motor response is usually flexion with a normal motor score expected of 3.

• Between 6 and 12 months, a normal infant makes noises, so normal verbal score expected is 3, an infant will usually locate pain, and so normal motor score expected is 4.

• Between 12 months and 2 years, recognizable words are expected, so normal verbal score expected is 4, and the infant will usually locate pain but not obeys commands, so normal motor score expected is 4.

• Between 2 and 5 years, recognizable words are expected with a normal verbal score expected of 4, and the infant will usually obeys commands, so normal motor score expected is 5.

• Children older than 5 years are orientated, aware of their location (home, hospital), so normal verbal score expected is 5.

In conclusion, PCS is extremely useful for all pediatric TBI, in perfectly connection with the age. Besides, this scale is very easy to work with. 

***2. Children’s Coma Score (CCS) (Raimondi & Hirschauer, 1984)[**4**]***

This scale evaluates, also, eyes opening response, verbal response and motor response to stimuli, but it is limited only for infants and toddlers. 

**Table:** 

Ocular response	Score
Pursuit	4
Extra ocular muscles (EOM) intact, reactive pupils	3
Fixed pupils or EOM impaired	2
Fixed pupils or EOM paralyzed	1
Verbal response	Score
Cries	3
Spontaneous respiration	2
Apnea	1
Motor response	Score
Flexes and extends	4
Withdraws from painful stimuli	3
Hypertonic	2
Flaccid	1

Maximum CCS assignable is 11, and minimal 3.[**4**] In conclusion, CCS is very useful for all pediatric TBI, in infants and toddlers. 

***3. Trauma Infant Neurological Score (TINS)[**5**]***

TINS is used for evaluation of TBI severity in infants and children under 3 years old, combines clinical and history elements: mechanism of trauma, orotraheal intubation on arrival, neurological exam, presence of subgaleal hematoma. 

**Table:** 

	Min/Max	0	1	2
Mechanism of trauma	1/2	-	Fall < 1 m or mild blow	Fall > l m or penetrating injury
Intubated on arrival	0/1	No	Yes	-
Alertness	0/2	Fully alert, but arousable	Decreased	Unconscious
Motor deficit	0/2	None	Lateralizing signs	No movement
Pupils	0/2	Reactive bilaterally	Anisocoria or non reactive pupil	Dilated and non reactive
Scalp injury	0/1	None	Subgaleal hematoma	-

Total score ranges from 1 to 10 points. TINS score over 2 indicates the need for a CT-scan examination. In conclusion, TINS is very useful in TBI in 0-3 year old children, because it evaluates mechanism of trauma, neurological and general status of the patient and scalp injury. Also, TINS reflects outcome of these patients (at 10 points the outcome is critical status).

***4. Glasgow Coma Scale (GCS)***

The level of consciousness in infants and young children with cranial traumas is quantified by GCS score, also used to grade the severity of brain injury.[**6**] GCS comprises three parameters: eye response, verbal response and motor response. GCS score is the sum of the assessment in each of the three categories: **GCS score = E + M + V**

The author shares the eye, verbal and motor response in preverbal children (0-1 year) and over 1 year, because there are important differences in connection with age. In preverbal children, the pediatric version of GCS is very rare used. The presentation of these parameters is given below.[**6**] The „grimace” alternative to verbal responses should be used in preverbal children. 

**Table:** 

Eyes opening/Ocular response:			
0-1 year	> 1 year	Score	
Spontaneously	Spontaneously	4	
To shout	To verbal command	3	
To pain	To pain	2	
No response	No response	1	
Motor response			
0-1 year	> 1 year	Score	
	Obeys command	6	
Localizes pain	Localizes pain	5	
Flexion withdrawal	Flexion withdrawal	4	
Flexion abnormal (decorticate)	Flexion abnormal (decorticate)	3	
Extension (decerebrate)	Extension (decerebrate)	2	
No response	No response	1	
Verbal response			
0-2 years	2-5 years	> 5 years	Score
Cries appropriately	Appropriate words	Oriented and converses	5
Cries	Inappropriate words	Disoriented and converses	4
Inappropriate crying or screaming	Screams	Inappropriate words, cries	3
Grunts	Grunts	Incomprehensible sounds	2
No response	No response	No response	1
The grimace response			
	Score		
Spontaneous normal facial activity	5		
Less than usual spontaneous response to touch stimuli	4		
Vigorous grimace to pain	3		
Mild grimace to pain	2		
No response to pain	1		

Intubated children are unable to speak and they are evaluated only with eye opening and motor response. The letter „T” is added to the score to indicate an intubated patient: the maximal GCS score is 10T and the minimum score is 2T. In conclusion, GCS score adapted from adults to infants and young children it is a pediatric version of GCS, but it is difficult to use in practical neuropediatric traumatology.

In this conditions, in our opinion, for children, are useful PCS, CCS and TINS, scales adapted for infants and toddlers.

***5. Liege Scale[**7**]***

Adding information on brainstem reflexes improves the prognostic precision of the Glasgow coma scale for patients with severe head injury. The Glasgow-Liege scale, improves the precision of prognosis, especially in head trauma patients with initial and complete LOC. 

**Table:** 

Brainstem reflexes	Scores
Fronto-orbicular reflex	5
Vertical oculocephalic or oculovestibular reflex	4
Pupillary light reflex	3
Horizontal oculocephalic or oculovestibular reflex	2
Oculocardiac reflex	1

## Neurotrauma pediatric outcome scales

In children we must use pediatric outcome scales, according to age. The most important neurotrauma pediatric outcome scales are: Glasgow Outcome Scale (GOS), modified Rankin score, KOSCHI (King’s Outcome Scale for Childhood Head Injury) score and Barthel Index. 

***Glasgow Outcome Scale (GOS) (Jennett & Bond, 1975) [**8**]***

**Table:** 

Score	Outcome		Description
1	Death	D	Death.
2	Vegetative state	VG	Patient exhibit no obvious cortical function.
3	Severe disability	SD	Conscious, but disabled. Patient depends upon other for daily support due to mental or physical disability or both.
4	Moderate disability	MD	Disabled, but independent. Patient is independent as far as daily life is concerned. The disabilities found include varying degrees of dysphasia, hemiparesis, ataxia, as well as intellectual and memory deficits and personality changes.
5	Good recovery	GR	Resumption of normal activities even though there may be minor neurological or physiological deficits.

GOS is the most common scale used to evaluate neurotrauma patients, it is very practical, it is very easy to work with, and it is well known by doctor of all specialties.

***Modified Rankin Score (1957) [**9**]***

**Table:** 

Score	Description
0	No symptoms at all
1	No significant disability despite symptoms; able to carry out all usual duties and activities
2	Slight disability; unable to carry out all previous activities, but able to look after own affairs without assistance
3	Moderate disability; requiring some help, but able to walk without assistance
4	Moderately severe disability; unable to walk without assistance and unable to attend to own bodily needs without assistance
5	Severe disability; bedridden, incontinent and requiring constant nursing care and attention
6	Dead

Rankin score was first described in vascular pathology, and extended afterwards for traumatic patients. It is more detailed than GOS, providing more information regarding the patient’s condition.

***KOSCHI Score (2001)[**10**]***

**Table:** 

Score	Description	Definition
1	Death	Death
2	Vegetative state	breathing spontaneously; no ability to communicate verbally or nonverbally or to respond to commands
3	Severe disability	a) some purposeful movement or ability to follow commands; may be conscious and able to communicate; unable to care for self b) exhibits high level of dependency but can assist with own care; fully conscious but with PTA
4	Moderate disability	a) mostly independent but requires supervision or help; has overt problems b) age appropriately independent but with residual learning/behavior problems or neurological sequels
5	Good recovery	a) HI resulted in new condition that does not affect well being or func-tioning b) complete recovery with no detectable sequels

KOSCHI score divides each outcome type of GOS into two subtypes, providing more accurate information about the patient’s status. 

***Barthel Index[**11**]***

Barthel index measures the patient's performance in 10 activities of daily life. The items can be divided into a group that is related to self-care (feeding, grooming, bathing, dressing, bowel and bladder care, and toilet use) and a group related to mobility (ambulation, transfers, and stair climbing). The maximal score is 100 if 5-point increments are used, indicating that the patient is fully independent in physical functioning. The lowest score is 0, representing a totally dependent bedridden state. Barthel Index is predictive for outcome following severe HI.

**Table:** 

Activity		Score
Feeding	unable	0
	needs help cutting, spreading butter, etc., or requires modified diet	5
	independent	10
Bathing	dependent	0
	independent (or in shower)	5
Grooming	needs to help with personal care	0
	independent face/hair/teeth/shaving (implements rovided)	5
Dressing	dependent	0
	needs help but can do about half unaided	5
	independent (including buttons, zips, laces, etc.)	10
Bowels	incontinent (or needs to be given enemas)	0
	occasional accident	5
	continent	10
Bladder	incontinent, or catheterized and unable to manage alone	0
	occasional accident	5
	continent	10
Toilet use	dependent	0
	needs some help, but can do something alone	5
	independent (on and off, dressing, wiping)	10
Transfers (bed to chair and back)	unable, no sitting balance	0
	major help (one or two people, physical), can sit	5
	minor help (verbal or physical)	10
	independent	15
Mobility (on level surfaces)	immobile or < 50 yards	
	wheelchair independent, including corners, > 50 yards	5
	walks with help of one person (verbal or physical) > 50 yards	10
	independent (but may use any aid; for example, stick) > 50 yards	15
Stairs	unable	0
	needs help (verbal, physical, carrying aid)	5
	independent	10

Barthel index is a large, exhaustive scale which evaluates patient’s integration into the family and society environment. It is also a dynamic and repeatable scale and is an accurate outcome evaluation method.

**Grading of traumatic brain injury**

Grading the severity of TBI in children should be done, as in adults, according to **GCS** score in: **minor head injury** (HI) (GCS score = 13-15), **moderate HI** (GCS score = 9-12) and **severe HI** (GCS score = 3-8). Each category has specific diagnosis, evaluation, management, different treatment strategies, complication, and particular outcome. 

***Head Injury Severity Scale (HISS)*** introduces two new criteria [**12**]:

• **Minimal TBI:** GCS score = 15 points, no loss of consciousness (LOC), no posttraumatic amnesia (PTA)

• **Mild TBI:** GCS score = 14 points, brief LOC under 5 minutes, PTA

• **Moderate TBI:** GCS score = 9-13 points, LOC over 5 minutes, focal neurological deficit

• **Severe TBI:** GCS score = 5-8 points

• **Critical TBI:** GCS score = 3-4 points

More accurate grading of TBI must also take into consideration other important criteria, too, like: mechanism of injury (e.g. the fall off a swing is a more aggressive mechanism of trauma, than fall from the same level, etc.), LOC, PTA, vomiting, and posttraumatic seizures.

TBI are classified as **closed or opened HI**. Penetrating head injury (PHI) must have a breach into dural layer that allow communication of endocranial structures with outside environment. PHI presents with craniocerebral wound, nasal or otic cerebrospinal fluid (CSF) leakage or pneumocephalus.

According to imagistic studies, CT-scan and MRI, head injuries are classified as **focal or diffuse lesions**. 

**Focal injuries** are well-defined, macroscopic lesions that lead to neurological dysfunctions due to local parenchyma changes or compression on surrounding structures, or cerebral herniation: focal contusion, laceration, hemorrhage and traumatic intracranial hematoma.

**Diffuse lesions** lead to alteration of consciousness with different duration: concussion, and diffuse axonal injuries (DAI). Concussion is a brief and sudden neuronal depolarization, with no anatomical changes on CT-scan; common clinical findings are disorientation, or short LOC. DAI are microscopically axonal damages, tissue tear hemorrhages, within the thalamus, basal ganglia, corpus callosum, superior cerebral peduncles, periventricular areas, and within the white matter the cerebral cortex. DAI are frequently encountered in severe TBI and clinical findings are prolonged, deep coma.

Correlation of clinical findings, severity of TBI, presence of neurological deficits, imagistic aspects of the lesions allow establishment of optimal management, therapeutically strategies and outcome possibilities.

## Minor head injury 

Almost 90% of pediatric HIs are minor. The child sustained minor HI (is alert, with a normal neurologic performance, and presenting inconstant vomiting. GCS score is 13-15 points. Local exam may show scalp lesions: epicranial hematoma, skin abrasions or lacerations. 

Grading in minor HI

• Grade 0 minor HI – no LOC, impact site localized pain, bruises, scalp abrasions, epicranial hematoma; CT-scan is not necessary, the patient is discharged home with instructions.

• Grade 0 with risk minor HI - no LOC, but the patients belong to the following categories: extreme age, history of neurosurgery, ventriculoperithoneal shunt, seizures, anticoagulant therapy, drugs or alcohol abuse (very rare in children); CT-scan is performed, and the patients is admitted for at least 24 hours.

• Grade 1 minor HI – LOC, PTA, persistent headache, vomiting, large scalp wounds; CT-scan must be performed within the first 6 hours after trauma, and the patient requires hospitalization even if the CT-scan, native and bone window, is normal.

• Grade 2 minor HI – GCS=13-14 points, LOC maximum 30 minutes, no focal deficits; the patient requires CT-scan and hospitalization.

Skull X-ray positive for a skull fracture, requires head CT-scan, native and bone window, and admission for observation of the child [**13**]. CT-scan can show a small size focal lesion, located within a noneloquent area, lacking of neurological signs. In this case, HI is not considered minor anymore. In that condition, the classification of HI, according to the severity should be done only after craniocerebral diagnostic imagery scan.

The child is discharged home but only after informed consent of parents. Both the parents and child must be aware of persistent or increased headache, vomiting, changes within level of consciousness, drowsiness, seizures (single or multiple posttraumatic seizures lasting more than 2 minutes) - situations in which they must contact immediately a neurosurgeon. Occurrence of any of these signs requires immediately neurosurgical reexamination of the child. CT-scan is mandatory in children with neurosurgical interventions and shunted patients. Also CT-scan is mandatory in all road accidents, passenger or pedestrian, and in child abuse. In these two situations CT-scan will be extended to cervical spine.

**Mild head injury**

According to HISS, a child with mild head injury (MHI) is has GCS of 14 points, brief LOC under 5 minutes, and PTA. MHI is of great importance in children, because of possibility of multiple posttraumatic neurobehavioral sequelles.[**14**] Children presenting with MHI may subsequently deteriorate and die from intracranial causes, situation known as „talk and die” syndrome.

We suggest performing a CT-scan in all children presenting with MHI, ever if they appear in perfect health, because of the risk of developing subsequent serious intracranial complications [**15**;**16**].

**Moderate head injury**

In moderate HI there is a history of trauma with LOC, changes in mental status, repeated vomiting, and focal neurological deficit. The child may present, even during examination, LOC or seizures. Local exam may show scalp lesions: epicranial hematoma or, more frequently, skin abrasions or lacerations. GCS score ranges between 9 to 12 points [**14**].

Immediate CT-scan and admission into a pediatric neurosurgical department are mandatory. Therapy is individualized according to age, type of injury, PCS, CCS, TINS and GCS scores, CT-scan and evolution.

Children with favorable outcome, with normal neurological exam, and normal CT-scan, can be discharged after few days’ hospitalization, and can be observed at home.

**Severe head injury**

The child with a severe HI is unconscious, often immediately after the injury, and has a GCS score between 3 and 8 points (comatose patient). Clinical exam of a posttraumatic unconsciousness child consists of assessment of vital signs, neurological exam, pupils’ size, and their reaction to light, brainstem reflexes, checking for cervical spinal cord injuries etc., and obtaining history of trauma.

Local cranial exam possible shows bruise, swelling or laceration on the scalp, raises suspicion of open or depressed skull injury or tense fontanelle, signs of basal skull fracture as hemotympanum, “raccoon’s eyes”, CSF leakage from the ear or nose, Battle’s sign and craniocerebral wound, etc.

A CT-scan, native and bone window, is performed immediately, and according to the neuroimaging result the child is admitted into the pediatric intensive care unit (PICU) or is taken directly into the operating room, where specific treatment is initiated [**17**]. It is better to extend CT-scan to cervical spine and in road traffic accidents to thoracic and abdominal area [**17**]. A normal CT-scan within 4 hours after the injury, does not rule out later complications, so repeating the investigation is necessary [**18**]. First and second CT-scans are useful as prognostic factor [**18**]. Intracranial lesions with high risk for progression and requiring surgery, such as extradural, subdural or intraparenchymal hematomas, must be observed by repeated CT-scan, even if no accompanying change in neurological status is noted [**19**]. On the other hand, intracranial lesions like subarachnoid hemorrhage, intraventricular hemorrhage, DAI and isolated skull fractures have small risk for progression, and CT-scan is recommended in cases with changes in neurological status [**19**]. Attention should be paid in children with severe HI, because most likely they may require repeated CT-scan examination. They should be given proper CT-scan examinations, enough for establishing the optimal therapeutic strategy, but the doctors must not exceed with this investigation, because of the risk radiation exposure. Radiation in infants and children increases lifetime risk cancer comparative with adults [**20**,-**22**,] and impairs cognitive function [**23**,]. But in spite of all risks, CT remains the gold-standard examination for children with cranial traumas. Protocols for indication of CT-scan examination were made [**24**,].

All these patients with severe HI are in comatose status and must be admitted in PICU with special facilities for infants, toddlers and children.

Predictive factors for poor outcome in severe HI are: ICP values at admission over ≥ 20 mmHg, presence of DAI, and low GCS at admission [**17**].

The context of a politrauma is aggravating the outcome [**17**]. Other factors influencing the outcome are prehospital care. 

## Conclusions 

Head injury in newborns, infants, toddlers and children has age-related specificity and anatomic particularities, for each of this period of development, comparative with adults. For this reason neurotrauma scales must be adapted, according to age. The authors present this thoroughgoing research on pediatric neurotrauma and outcome scales, concordant to international data, to help study of traumatic brain injury by pediatric neurosurgeons, neurologists and intensive care-anesthesiologists.

Pediatric neurotrauma and outcome scales are compulsory for assessment of children presenting with HI. They establish criteria for admission and for short and long-term outcome.

The authors stress on grading the severity of traumatic brain injury, according to the latest literature data.

## Abbreviations 

CCS Children’s Coma Score

CNS central nervous system

CSF cerebrospinal fluid

DAI diffuse axonal injuries

EOM extraocular muscles 

GCS Glasgow Coma Scale

GOS Glasgow Outcome Scale

HI head injury

HISS Head Injury Severity Scale 

KOSCHI King’s Outcome Scale for Childhood Head Injury 

LOC loss of consciousness 

MHI mild head injury 

MISS Modified Injury Severity Scale

PCS Pediatric Coma Scale

PHI penetrating head injury

PICU pediatric intensive care unit 

PTA posttraumatic amnesia

TBI traumatic brain injury

TINS Trauma Infant Neurological Score 
